# Potential Roles of Endoplasmic Reticulum Stress and Cellular Proteins Implicated in Diabesity

**DOI:** 10.1155/2021/8830880

**Published:** 2021-04-27

**Authors:** Sagir Mustapha, Mustapha Mohammed, Ahmad Khusairi Azemi, Ismaeel Yunusa, Aishatu Shehu, Lukman Mustapha, Yusuf Wada, Mubarak Hussaini Ahmad, Wan Amir Nizam Wan Ahmad, Aida Hanum Ghulam Rasool, Siti Safiah Mokhtar

**Affiliations:** ^1^Department of Pharmacology, School of Medical Sciences, Universiti Sains Malaysia, 16150 Kota Bharu, Kelantan, Malaysia; ^2^Department of Pharmacology and Therapeutics, Ahmadu Bello University Zaria, Kaduna, Nigeria; ^3^School of Pharmaceutical Sciences, Universiti Sains Malaysia, 11800 Penang, Pulau Pinang, Malaysia; ^4^Department of Clinical Pharmacy and Pharmacy Practice, Ahmadu Bello University Zaria, Kaduna, Nigeria; ^5^Department of Clinical Pharmacy and Outcomes Sciences, University of South Carolina, College of Pharmacy, Columbia, SC, USA; ^6^Department of Pharmaceutical and Medicinal Chemistry, Kaduna State University, Kaduna, Nigeria; ^7^Department of Medical Microbiology and Parasitology, School of Medical Sciences, Universiti Sains Malaysia, 16150 Kota Bharu, Kelantan, Malaysia; ^8^Department of Zoology, Ahmadu Bello University Zaria, Kaduna, Nigeria; ^9^School of Pharmacy Technician, Aminu Dabo College of Health Sciences and Technology, Kano, Nigeria; ^10^Biomedicine Programme, School of Health Sciences, Universiti Sains Malaysia, 16150 Kota Bharu, Kelantan, Malaysia; ^11^Hospital Universiti Sains Malaysia, 16150 Kota Bharu, Kelantan, Malaysia

## Abstract

The role of the endoplasmic reticulum (ER) has evolved from protein synthesis, processing, and other secretory pathways to forming a foundation for lipid biosynthesis and other metabolic functions. Maintaining ER homeostasis is essential for normal cellular function and survival. An imbalance in the ER implied stressful conditions such as metabolic distress, which activates a protective process called unfolded protein response (UPR). This response is activated through some canonical branches of ER stress, i.e., the protein kinase RNA-like endoplasmic reticulum kinase (PERK), inositol-requiring enzyme 1*α* (IRE1*α*), and activating transcription factor 6 (ATF6). Therefore, chronic hyperglycemia, hyperinsulinemia, increased proinflammatory cytokines, and free fatty acids (FFAs) found in diabesity (a pathophysiological link between obesity and diabetes) could lead to ER stress. However, limited data exist regarding ER stress and its association with diabesity, particularly the implicated proteins and molecular mechanisms. Thus, this review highlights the role of ER stress in relation to some proteins involved in diabesity pathogenesis and provides insight into possible pathways that could serve as novel targets for therapeutic intervention.

## 1. Introduction

Diabetes is a complex condition associated with a high amount of glucose in the blood resulting from an absolute or relative lack of insulin [1]. Its increasing global prevalence has been attributed mainly to the pandemic of obesity and sedentary lifestyles [2, 3]. Obesity is a significant risk factor for the development of diabetes [4]. In turn, obesity may coexist with diabetes, a condition referred to as diabesity [5]. About 30% of obese patients are at risk of advancing into diabesity, owing to decreased tyrosine phosphorylation levels on insulin receptor substrate 1 (IRS-1) [6, 7]. Usually, the insulin activates the insulin receptor via dimerization and transautophosphorylation, which phosphorylate the tyrosine residues site on IRS-1 and IRS-2, leading to the activation and recruitment of other cellular components needed for an insulin signaling cascade, such as phosphatidylinositol 3-kinase/protein kinase B (PI3k/Akt/eNOS).

Diabesity as a metabolic disorder that presents with hyperglycemia and hyperlipidemia, leading to endoplasmic reticulum (ER) stress and ultimately contributing to insulin resistance. The ER structure is the central station for protein synthesis and serves other metabolic functions [8]. The cellular activation of IRS-1 by insulin receptors stimulates sustained cell growth and an antiapoptotic response, whereas insulin resistance from ER stress leads to inhibition of the IRS-1 functions [9]. ER stress results in serine phosphorylation of IRS-1 via c Jun N terminal kinases (JNK), leading to insulin resistance. Insulin resistance stimulates serine/threonine kinases that phosphorylate IRS-1, thereby inhibiting its normal functions. Several studies have proposed mediators such as endothelin-1, free fatty acids (FFA), tumor necrosis alpha (TNF*α*), amino acids, angiotensin II (ang II), and hyperinsulinemia as catalysts for insulin resistance [10–17]. However, little is known about the relationship between ER stress and proteins such as endothelial nitric oxide synthase (eNOS), endothelium-derived endothelin-1 (ET-1), insulin receptor substrate (IRS-1 and IRS-2), nicotinamide adenine dinucleotide phosphate (NAD(P)H) oxidase, and caveolin 1 (Cav-1), all of which are implicated in diabesity.

At the cellular level, a drop in glucose uptake as a result of a decline in IRS-1 tyrosine phosphorylation and phosphatidylinositol 3-kinase (PI3k) stimulation in animal models leads to high levels of glucose in the blood, which initiate ER stress [6, 18]. In the pathogenesis of diabesity, hyperglycemia and hyperinsulinemia cause more glucose to be oxidized via the pentose phosphate pathway (PPP), creating a massive NADPH electron donor to fuel superoxide anion production [19]. A large amount of superoxide anion in the cell may lead to ER stress [20]. ER stress, in turn, may affect normal physiological conditions in the cell.

Several mechanisms of diabesity in relation to ER stress have been reported. It is believed that ER stress leads to abnormal cell signaling in response to insulin, causing insulin resistance, as seen in diabesity. To date, no study has identified cellular proteins implicated in ER stress signaling pathways in diabesity. Therefore, this review is aimed at highlighting the roles of ER stress and its relationship with some cellular proteins implicated in diabesity, such as eNOS, ET-1, IRS-1 and IRS-2, NAD(P)H oxidase, and Cav-1.

## 2. The Endoplasmic Reticulum (ER)

The ER was first discovered in cultured mice fibroblast and later established in all eukaryotic cells, except matured red blood cells [21]. Borgese et al. [22] later reported the existence of smooth ER and rough ER. Shibata et al. [23] proposed a new concept that grouped ER into a membrane structure. The ER's architectural nature is organized into a nuclear envelope, sheet-like cisternae, and a polygonal array of tubules connected by three-way junctions [23]. The ER is the central station for protein synthesis, protein secretions, and other cellular metabolic functions [8]. It also serves as a signaling pathway between the cell cytosol and the nucleus [24]. Factors such as an oxidative environment, folding enzymes, chaperones, and a high amount of Ca^2+^ are needed for effective ER function [25]. Almanza et al. [26] reported that ER has advanced from a major role in protein synthesis to a foundation for metabolic functions. It is believed that the level of glucose in the body has a profound effect on ER stability, leading to a pathway activation called unfolded protein response (UPR). UPR acts via coping mechanisms such as enlarging the ER's size, among other responses, for cell survival [27]. When these coping mechanisms fail, a condition called ER stress results [28].

The mitochondria-associated ER membrane (MAM) is a structure that exists between the ER and mitochondria. The MAM plays a crucial role in Ca^2+^ stability [29]. Endolysosomal system circulation also helps maintain cellular homeostasis since it controls the internalization and processing of a broad range of integral proteins, such as signaling receptors, adhesion molecules, nutrient transporters, and lysosomal hydrolase receptors [30]. Morphology-1 comprises new mitochondria-vacuole membrane contact sites (MCSs) responsible for tethering protein for ER-vacuole/lysosome, which precisely locates nuclear ER-vacuole junctions. Mitochondrial-mediated interaction between ER and endolysosomal system circulation and morphology-1 complexes suggests ER participation in autophagy [31]. The omegasome (a structure in the ER) interacts with the phagophore (a tiny, nanomembrane sac-like structure), resulting in the formation of autophagosomes, which play an essential role in the cell degradation pathway [32, 33]. Thus, it is imperative to understand the in-depth relationship between ER and other cellular organelles, such as mitochondria, lysosomes, Golgi body, peroxisomes, endosomes, and plasma membrane, to gain insights that may pave the way to optimal management of diabesity.

## 3. The ER Stress Mechanism

ER homeostasis plays a critical role in cellular function and growth, while ER stress causes a physiopathological process that can lead to diabetes, obesity, cancer, and diabesity [34–36]. ER stress sets in when the ER condition is unstable because of unfolded proteins that exceed its handling ability [34]. ER stress in human tissues has also been shown to be caused by hyperglycemia and hyperinsulinemia [37]. Hyperglycemia and hyperinsulinemia are associated with enhanced protein production and posttranslational protein modifications such as ubiquitinations, indicating an amplified misfolded or unfolded protein, a significant source of ER stress [37, 38]. Prior to ER stress, cells primarily adjust to misfolded protein overload in the ER lumen by increasing the amount of molecular chaperones, called binding immunoglobulin protein (BiP) [39]. BiP is a molecular chaperone found in the ER lumen only. In the absence of stress, BiP is primarily known to bind to the three kinds of ER arm sensors, namely, inositol-requiring kinase 1 (IRE1), PKR-like ER kinase (PERK), and activating transcription factor 6 (ATF6) transmembrane, thereby avoiding ER stress initiation [34, 40], and BiP is known to be sensitive to glucose concentration in the cell [41, 42]. The literature contains no clear indication of which of the three above-named ER arm sensors is activated first. However, Hu et al. [43] reported that the first pathway activated among the three ER protein membranes is PERK. When ER stress has been initiated due to unfolded protein, the three-arm sensors are usually all affected [27]. The molecular mechanisms underlying ER stress are not clearly understood in association with diabesity.

IRE1 is expressed in two forms, either IRE1*β* or IRE1*α*. IRE1*β* is mainly found in the epithelium of the gastrointestinal tract [44], while IRE1*α* is pervasively present in all cells but particularly prolific in the eukaryotic cells and is exceptionally well preserved [27]. Usually, IRE1 binds to BiP in the ER lumen to form a nonactive complex. A high concentration of misfolded proteins in the luminal area of ER leads to kinase activities that cause IRE1*α* activation to elicit its RNase activity during ER stress. IRE1*α*, type 1 ER transmembrane protein, undergoes kinase/endoribonuclease, oligomerization, and autophosphorylation. IRE-1 has a novel endoribonuclease function that contributes to its substrate, the 26-nucleotide sequence X box-binding protein 1 (XBP1) mRNA, being excised and spliced. Spliced XBP1 results in the overexpression of genes responsible for unfolded protein degradation and ER protein translocation, folding, and secretion [45, 46]. Also, spliced XBP1 translocates to the promoter region of the ER stress-mediated response and binds to it, enabling it to control many genes associated with UPR that are crucial in stimulating the degradation of dysregulated cells [47, 48]. IRE1*α* can also break precursor microRNAs (miRNAs) or a small collection of mRNAs in a process called regulated IRE1-dependent decay (RIDD). RIDD may lead to the degradation of mRNA and prevent protein load. RIDD has been found to control multiple signaling pathways on the ER, including selectively cleaving mRNAs [49, 50]. Recently, IRE1*α* was discovered to form complexes with the machinery of translocation and translational parts, such as transfer RNA, signal recognition particle RNA, and ribosomal RNA. However, the biological essence of these interactions remains to be elucidated. IRE1*α* interactions with RNA are unknown, but its biological activities remain to be seen. The crosstalk between IRE1*α* and some noncanonical mediators such as mitogen-activated protein kinase (MAPK) and macroautophagy is of great interest. Also, IRE-1 interacts with tumor necrosis factor *α* receptor-associated factor 2 (TRAF2) to activate inflammatory response-related protein kinases and cellular apoptosis, such as apoptosis signal-regulating kinase 1 (ASK1) and triggers the activation of JNK [28]. JNK is activated in the mitochondria and facilitates the phosphorylation of proapoptotic proteins, leading to the initiation of cell death and inhibiting antiapoptotic proteins [51].

There are three physiological variants of IRE1*α*, i.e., monomeric, dimeric, and multimeric forms. The monomeric form is bound to the BiP at the N-terminal luminal domain (NLD) to form an inactive complex, while the dimeric and multimeric are active forms. It is crucial to understand IRE1 from a broader perspective as an ER stress arm-sensor, not limited to mechanisms and structure.

When PERK is dissociated from BiP, it will undergo dimerization and autophosphorylation. The phosphorylated PERK stimulates and phosphorylates eukaryotic initiation factor 2*α* (peIF2*α*) [27]. peIF2*α* leads to a downstream signaling cascade that attenuates protein production and transcriptional factors such as activation transcription factor-4 (ATF-4) and CCAAT/enhancer-binding protein (C/EBP) homologous protein (CHOP). These transcriptions promote genes responsible for survival [52, 53]. The downstream signaling cascade is a reversible process that limits the entry of newly formed proteins into the ER lumen. It also promotes maintaining ER homeostasis and proper protein folding, as well as assembling. PERK also takes part in tethering the association between the ER and mitochondria, which may involve ROS production [54]. The initial activation of activating transcription factor-4 (ATF-4) is for cell survival during UPR. The ATF-4 is involved in negative feedback to dephosphorylate eIF2*α* via protein phosphatase 1 (PP1) regulatory subunit growth arrest and DNA damage-inducible protein (GADD34). The GADD34 and the constitutive repressor of eIF2*α* phosphorylation (CReP) are essential agents in restoring normal protein production via PERK, as the cell has been relieved of ER stress. ATF-4 also takes part in the dephosphorylation of eIF2 via a negative feedback mechanism as protein production is being restored (using protein phosphatase 1 as a subunit of GADD34).

During chronic ER stress, PERK activates the downstream signaling of eIF2/ATF-4/CHOP, which alters the translational and transcriptional process, resulting in enhanced activation of nuclear factor-kappa B (NF-*κ*B) [55]. Therefore, activation of NF-*κ*B will lead to the elimination of the damaged cells. Cells that are deficient in PERK or phosphorylated eIF2*α* may increase the accumulation of misfolded or unfolded proteins in the ER lumen [25]. Several studies have demonstrated the relationship between ER stress and PERK. Elouil et al. [56] and Hou et al. [57] reported that prolonged hyperglycemia for more than 18 hours leads to PERK activation, thereby causing ER stress. However, Gomez et al. [58] reported that exposure to lower glucose levels and a shorter time is likely to facilitate PERK activation, resulting in the phosphorylation of eIF2*α*.

The ATF6p90, as the last arm sensor of ER stress, is a transmembrane transcriptional factor with both C terminal and N terminal domains [59]. It has two forms of mammal genes, namely, ATF6*α* and ATF6*β* [60]. During ER stress, there is an upsurge in the aggregation of misfolded proteins in the ER's lumen that leads to BiP detachment from ATF-6p90. When ATP6p90 reaches the Golgi apparatus, it is then cleaved via the protease site 1 and protease site 2 to discharge ATP6p50 containing basic leucine zipper, transcription activation domain, nuclear localization signals, and DNA binding domain. The nucleus receives this processed ATF-6p50 from the Golgi apparatus and induces the gene expression [61, 62]. Although ATF6p50 and XBP1s are parallel to each other, they intersect in their downstream signaling cascade to control gene transcription, such as endoplasmic reticulum-associated protein degradation (ERAD), enhanced protein folding, secretion and maturation, and ER chaperones [63, 64]. When the UPR fails to free the cell from ER stress, the cell will prepare for cell death by CHOP activation via ATF-6. For example, studies conducted by Wu et al. [65] and Yamamoto et al. [36] show that mice using ATF-6 inducers without ATF-6 had amplified ER stress. Also, it is worth noting that there are other basic leucine zipper transcription factors in the ER, such as cyclic adenosine monophosphate- (cAMP-) responsive element-binding protein H (CREB-H), cAMP-responsive element-binding protein-4 (CREB4), also known as Luman, and old astrocyte specifically induced substance (OASIS), which may be engaged during ER stress signaling [66]. This indicates how complex the ER stress signaling cascade is in association with other noncanonical pathways.

Therefore, understanding the complex mechanisms involving the three ER arm sensors and their interconnections is necessary if diseases such as diabesity are managed. It is crucial to establish which of the arm proteins is activated first, at what point, and the interswitch during ER stress. It is also essential to identify the impact of noncanonical pathways in diabesity.

## 4. Diabesity

**Figure 1:**
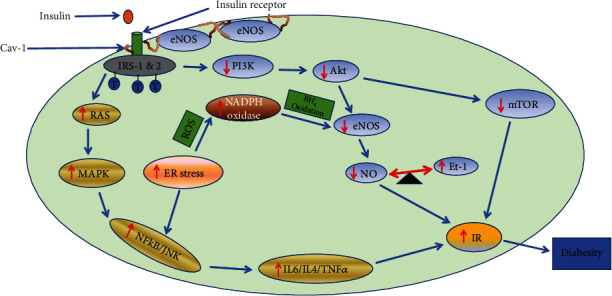
Relationship between insulin impairment, ER stress, and diabesity. JNK: c-Jun N-terminal kinase; NF-*κ*B: nuclear factor-kappa B; IR: insulin resistance; NO: nitric oxide; Akt: protein kinase B; MAPK: mitogen activated protein kinase; mTOR: mammalian target of rapamycin; PI3K: phosphoinositide-3-kinase; eNOS: endothelial nitric oxide synthase; IRS-1 and 2: insulin receptor substrate; ER stress: endoplasmic reticulum stress; IL6; interleukin-6; IL-4: interleukin-4; TNF*α*: tumour necrosis factor *α*; ROS; reactive oxygen species; BH_4_: tetrahydrobiopterin; NAD(P)H: nicotinamide adenine dinucleotide phosphate oxidase; *Cav-1*: *caveolin*-*1*; *Et-1*: *endothelin*-*1*.

Diabesity or obese diabetes occurs in subjects with obesity, who then develop type 2 diabetes [35]. Sims et al. [67] were the first investigators to coin the word diabesity in 1973, recognizing the cooccurrence of diabetes and obesity. The prevalence of obesity is increasing globally, as is that of type 2 diabetes. Diabesity exhibits common features in diabetes and obesity (such as adipose tissue, skeletal muscle, vascular, endothelial cell, and liver dysfunctions). Diabesity is associated with the disruption of metabolic cell signaling pathways and attenuated insulin signaling, that is, insulin dysfunction, which raises the risk of type 2 diabetes [68]. Diabesity, type 2 diabetes, and obesity alter many metabolic cascades, such as mitogen-activated protein kinase (MAPK), mammalian target of rapamycin (mTOR), and phosphoinositide-3-kinase/protein kinase B (PI3K/Akt), which has a systemic effect [69]. The attenuation of the above signaling cascade presents a condition called insulin dysfunction. Insulin dysfunction is also one of the main symptoms of obesity and diabesity. ER stress has been identified as one of the molecular mechanisms linked to insulin dysfunction [70]. ER stress has been seen in patients with diabesity, obesity, and type 2 diabetes, with significant effects on skeletal muscles, the fetoplacental vascular endothelium, visceral adipose tissue, and the liver. ER stress initiation via IRE-1*α* mediates stimulation of JNK and inhibitory kappa B kinase (IKK) [71]. The activation of JNK mediates the impairment of insulin via the serine phosphorylation of insulin receptor substrate (IRS) [72]. The end product of these metabolic cascades is called diabesity. The relationship between impaired insulin, ER stress, and diabesity is shown in Figure 1.

## 5. Cellular Proteins Implicated in Diabesity

Cellular proteins are macromolecules that are essential in many cellular functions in a living system. These proteins are involved in daily cellular activities, regulations, operations, and structures, one function being to help maintain the hemodynamic and structural integrity of a cell. Some cellular proteins affected by the physiopathology of diabesity include eNOS, ET-1, IRS-1, NADPH oxidase, and Cav-1. eNOS, as a cellular protein or enzyme, is essential for the production of nitric oxide (NO), while ET-1 is responsible for the vasoconstriction effect on the blood vessels. Also, IRS-1 plays an essential role in the insulin signaling pathways; meanwhile, NAD(P)H oxidase is responsible for producing reactive oxygen species that participate in the protein folding process. Cav-1 is the site for diverse signaling mechanisms, which could be interrupted due to ER stress.

## 6. ER Stress and Endothelial Nitric Oxide Synthase (eNOS)

For decades, the endothelium was viewed first as an inert barrier that coats all blood vessels along the vasculature. Extensive studies led to a breakthrough in understanding its dynamic roles and its function in sustaining cardiovascular stability. The endothelium is currently identified as a fundamental unit of the endocrine organ that possesses various metabolic, immunological, and secretory features rather than as an inert barrier. The endothelium begins from the heart and runs through the blood vessel walls in the body. Also, structurally endothelium-derived relaxing factor (EDRF), in the form of nitric oxide (NO) or a nitrogen oxide-containing compound, plays a crucial role in maintaining homeostasis in the vasculature and producing an antithrombotic effect. The total formation of NO in mice has been documented to be approximately 0.2 mmol/kg/day, of which nearly 70% is obtained from eNOS [73]. The NO synthesis rate in humans is approximately 0.9 *μ*mol/kg/h, while in Wistar rats, it is about 0.33–0.85 *μ*mol/kg/h [74–77]. NO generation is achieved through endothelial nitric oxide synthase (eNOS) [78].

eNOS consists of two similar subunits that exist as a homodimer structurally. The eNOS subunits contain the oxygenase domain and the reductase domain. The reductase domain has a binding site for the following cofactors: flavin mononucleotide (FMN), NADPH, and flavin adenine dinucleotide (FAD). In contrast, the oxygenase domain has binding sites for L-arginine, heme, and tetrahydrobiopterin (BH_4_). The calmodulin (CaM) binding domain serves to link the oxygenase domain and the reductase domain. The CaM binding domain plays a potential role in both functions and in maintaining the structure of eNOS. For eNOS to function under normal physiological conditions, the reductase domain and the oxygenase domain must come together in a homodimer in the presence of the cofactors (CaM, BH_4_, and heme) [79, 80]. The activation of eNOS could be calcium-dependent or calcium-independent (G protein, shear stress, and cyclic strain) [81–83].

The eNOS mechanisms are highly complex and can be divided into protein and genetic levels. The eNOS protein mechanism involves phosphorylation by Akt, CaM complex formation, transfer of electrons from the reductase domain to the oxygenase domain, and the translocation of eNOS. In the resting state, eNOS is attached to Cav-1 at the cell membrane. Cav-1 is bound to both the reductase domain and the oxygenase domain to prevent the heme iron from being in contact with eNOS, hindering electron movement from the reductase domain. It has been reported that eNOS and Cav-1 interact with two new proteins, i.e., eNOS traffic inducer (NOSTRIN) and eNOS interacting protein (NOSIP), to form a ternary complex. Such a complex triggers eNOS relocation from the cell membrane and significantly decreases eNOS activity [84, 85]. In response to various agonists such as shear pressure and bradykinin or acetylcholine (Ach), intracellular Ca^2+^ concentrations may increase. This causes Cav-1 to dissociate from eNOS, enabling CaM and heat shock protein 90 (hsp90) to bind to eNOS [86]. The binding of CaM to eNOS allows Akt to phosphorylate eNOS in both human and animal models at serine^1177^ and serine^1179^, respectively, while at the same time, Akt causes dephosphorylation at the inhibitory site of eNOS at threonine^495^ (Thr^495^) [83, 87]. When Akt phosphorylates eNOS at serine, NO production starts with amino acid L-arginine oxidation to generate NO and L-citrulline [88]. Akt often exists inactively in the cytoplasm; its activation and phosphorylation of eNOS are caused by translocation of Akt to the cell membrane [89]. The phosphatidylinositol-3 kinase pathways (IRS/PI3K/Akt) directly regulate this, Akt being recruited to the cell membrane and phosphorylated by PI3K [90]. It is important to note that increased intracellular Ca^2+^ concentration is needed for sufficient eNOS activation and NO bioavailability. This process is known as calcium-dependent eNOS phosphorylation. Bradykinin and 5-hydroxytryptamine (5-HT) interact with plasma membrane receptors, resulting in increased cytoplasmic Ca^2+^ and CaM activation [91].

The expression and stability of eNOS genes are linked to eNOS behavior at the genetic level. A substantial number of binding sites for transcription factors are included in the eNOS promoter region, comprising the endothelin family, neurofibromin 1 (NF-1), activator protein-1 (AP-1), nuclear factor-*κ*B (NF-*κ*B), and activator protein-2 (AP-2). These complexes in the transcription factor can control eNOS expression. It is now known that various factors affect eNOS expression, among them are estrogen, exercise, and hypoxia [92–94].

**Figure 2:**
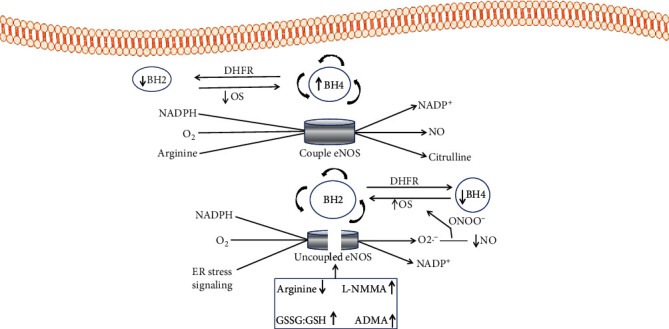
eNOS, NADPH, and ER stress consequential generation of NO and superoxide anion from coupled and uncoupled NOS. eNOS requires substrates such as oxygen, BH_4_ cofactors, L-arginine, NADPH, FMN, and heme. In the physiological circumstances, the availability of BH_4_ is sustained by guanosine triphosphate (GTP), such that the rate-limiting step is catalyzed by GTP cyclohydrolase I (GTPCH). The enzyme dihydrofolate reductase (DHFR) mediates the reprocessing of BH_2_ to produce BH_4_ as the primary nonenzymatic oxidation. eNOS uncoupling leads to the generation of superoxide anion. The superoxide anion produced results in decreased bioavailability of BH_4_. As such, superoxide anions generated owing to uncoupled eNOS reacts with NO to produce ONOO-, a highly reactive anion that quickly oxidizes BH_4_. Furthermore, self-propagating oxidative stress can stabilize eNOS uncoupling. Other demonstrated mechanisms that enhance the uncoupling of eNOS include high concentrations of endogenous NO synthase inhibitors, increased levels of oxidized glutathione relative to decreased glutathione, and reduced availability of arginine. BH2: dihydrobiopterin; BH4: tetrahydrobiopterin; DHFR: dihydrofolate reductase; OS: oxidative stress; NO: nitric oxide; eNOS: endothelial nitric oxide synthase; NADPH: nicotinamide adenine dinucleotide phosphate; ONOO-: peroxynitrite; L-NMMA: NG-monomethyl-L-arginine; ADMA: asymmetric dimethylarginine; GSSG: oxidized glutathione; GSH: reduced glutathione.

In pathological conditions such as diabetes, there is reduced bioavailability of BH_4_ and NADPH [95]. NAD(P)H oxidase is more active in diabesity than in normal physiological conditions, owing to hyperlipidemia, cytokines, ang II, and hyperglycemia [96]. The increased NAD(P)H oxidase activity results in the oxidation of BH_4_, a cofactor of eNOS, and the production of reactive oxygen species (ROS) [97]. ROS production by NAD(P)H oxidase may amplify ER stress [98]. The association between eNOS and ER stress may be due to chronic hyperglycemia, which increases oxidative stress [99]. ER stress signaling and increased NAD(P)H oxidase may be the initiators of eNOS uncoupling because of decreased NO bioavailability and amplified ROS generation [100, 101], as depicted in Figure 2. Hyperglycemia wholly or partially leads to increased oxidative stress, resulting in endothelial dysfunction [102]. Also, eNOS expression decreases when protein kinase C (PKC) is activated because of chronic hyperglycemia, which similarly in diabesity enhances the stimulation of NAD(P)H oxidase [103–105].

The dysfunction of endothelial cells occurs via eNOS uncoupling and increased ER stress. A study by Mokhtar et al. [106] reported decreased eNOS expression in the diabetic rat model; such a reduction could likely be due to ER stress. The high amount of glucose in the blood prompts the endothelial cell to initiate ER stress [107], which may be due to diabesity. First and foremost, it must be noted that ER stress contributes to insulin dysfunction, which leads to an enhanced progression of hyperglycemia in the blood vessels. The result of insulin dysfunction affects insulin signaling pathways (IRS/PI3K/Akt), which decreases the availability of Akt and in turn results in a small amount of NO because of eNOS uncoupling. Also, chronic hyperglycemia causes eNOS uncoupling, leading to endothelial dysfunction via many unidentified pathways. Thus, identifying these pathways can serve as a first step in preventing or reducing morbidity and mortality associated with diabesity.

## 7. ER Stress and Endothelium-Derived Endothelin-1 (ET-1)

Endothelin consists of three family isoforms (ET-1, ET-2, and ET-3), of which ET-1 is the most widely distributed member of the family [108]. ET-1 is recognized as an endothelium-derived contracting factor (EDCF) [109]. It is produced by many types of cells and contains 21-amino acid peptides [110]. ET-1 has a half-life of around 1 min in a healthy individual [111]. It is involved in the pathogenesis of many diseases, including type 1 diabetes, diabesity, hypertension, fibrosis, and atherosclerosis [112]. Endothelin receptors (ETRs) are G protein-coupled receptors comprising endothelin A receptor (ET_A_R) and endothelin B receptor (ET_B_R), which demonstrate opposite actions in cardiovascular conditions [113]. ET_A_R is found on the smooth muscle cells and mediates vasoconstriction. It also induces proliferative responses linked with type 1 diabetes, diabesity, hypertension, and other related cardiovascular diseases. However, ET_B_R is found on the vascular endothelial cells and mediates vasodilation [112]. It plays an essential role as a clearance receptor for the ET-1 in the system. The two ETRs are implicated in the development of many diseases, including diabesity. ET-1 is known to stimulate proteins associated with many signaling pathways, such as PI3K, NFkB, MAPK, phospholipases, beta-catenin, hypoxia-inducible factor 1 alpha, protein kinase C, RHO, and protein kinase A [114].

Vasoconstrictors enhance the production of ET-1, which results in increased vasoreactivity in response to serotonin, norepinephrine (NE), and ang II [115]. Typically, insulin binds to the insulin receptor (IR) and activates the receptor via dimerization and transautophosphorylation of the tyrosine residue. IR phosphorylation leads to activation and phosphorylation of the IRS family (IRS-1 and IRS-2). The Src homology 2 domain docks on the IRS-1, bound by growth factor receptor-bound protein 2(Grb-2) [116]. Grb-2 serves to stimulate the preassociated guanine nucleotide exchange factor Son of Sevenless (SOS). The SOS protein activates Ras-bound guanosine diphosphate (GDP) to Ras-bound guanosine triphosphate (GTP). Ras becomes more active with GTP, which activates and phosphorylates a cytoplasmic protein called Raf (MAPKKK). The activation of Raf initiate kinase signaling by, for example, MAPKK and MAPK. An MAPK insulin signaling cascade regulates actions associated with differentiation, growth, mitogenesis, ET-1 [117, 118], plasminogen activator inhibitor type 1 (PAI-1), vascular cell adhesion molecule-1 (VCAM-1), and E-selectin [119]. It is important to note that impairment of IRS/PI3K/Akt signaling pathways results in overexpression of ET-1, E-selectin, and VCAM-1 and decreases the bioavailability of NO owing to the overactivation of MAPK signaling pathways [119]. The consequences for the endothelial function of both insulin dysfunction and hyperinsulinemia due to diabesity are more severe than the individual effects.

The overexpression of ET-1 in the endothelium causes an amplified expression of genes responsible for lipid production in the vascular cells [120]. This could lead to an overload of misfolded and unfolded proteins in the ER lumen, resulting in ER stress. Sustained periods of cellular stress may cause a build-up of misfolded proteins in the ER lumen, exceeding its protein-folding capacity and thence leading to activation of a coping signaling mechanism known as UPR [121–123]. Enhanced levels of ET-1 are a sign of pathology in many diseases that trigger processes such as ER stress, inflammation, and oxidative stress. Physiological messengers like ROS are essential in maintaining homeostasis in vasculatures, but their increased generation may contribute to ER stress progression, leading to diabesity. A study by Mian et al. [124] demonstrated that ET-1 is overexpressed in the endothelium of mice, owing to impairment of IRS/PI3K/Akt pathways with an increase in voltage-dependent potassium channel activity. Also, ET-1 activates ET_B_R, which in turn stimulates phospholipase C (PLC) and inositol 1,4,5-trisphosphate (IP3) in the ER to discharge Ca^2+^. These actions initiate ER stress in the placenta tissue [125]. ET-1 also causes the overexpression of molecular chaperones (GRP78 and GRP94) and enhanced phosphorylation of eIF2*α*, ER stress indicators [125]. A study by Wang et al. [126] on mice showed an increase in ER stress biomarkers (GRP78 and CHOP). Therefore, it is suggested that ER stress is activated by ET-1 in diseases. In diabesity, ET-1 is likely a potent initiator of ER stress via a dual action by disrupting the Ca^2+^ER balance. In some studies, it was found that ET-1 is increased in diabetic patients compared to controls [127, 128].

## 8. ER Stress and Inhibition of the Insulin Receptor Substrate

Nutrient overload may be implicated in the etiology of diabetes, diabesity, and insulin resistance [35, 129]. Resistance to insulin causes dyslipidemia, hyperinsulinemia, hypertension, and hyperglycemia [35]. Many factors lead to insulin resistance, including IRS-1 and IRS-2 [130–133]. Insulin signaling in physiological and pathological circumstances is modulated by the insulin receptor substrate (IRS) family. IRS has an amino and carboxyl group domain that contains the serine/threonine and tyrosine sites for phosphorylation [134]. The concentration of insulin required to activate and phosphorylate PI3K/Akt/eNOS pathways is less than the concentration required to phosphorylate the Ras/MAPK pathway [135]. Usually, insulin activates the insulin receptor via dimerization and transautophosphorylation of the insulin receptor. This process leads to the recruitment and phosphorylation of tyrosine residues on IRS-1 and IRS-2, which activate and recruit Src homology domain proteins. The Src homology domain provides a docking site for Grb-2 and SOS [130]. Grb-2 results in the activation and recruitment of PI3K, which leads to a downstream signaling cascade of insulin. The duration or strength of these mechanisms is influenced by many factors, including the stability of the insulin isoform receptors (IRA and IRB) or postreceptor activities such as insulin signaling cascade (IRS/PI3K/Akt/eNOS) [136]. Other regulatory proteins and signaling cascades can have a negative impact on the insulin signaling pathway by heightening insulin resistance, for example, phosphatidylcholine transfer protein [137], the Lin28/Let-7 axis [138], muscle-specific mitsugumin 53/tripartite motif 72 (MG53/TRIM72) [139], Grb10 [140], and the cullin RING E3 ubiquitin ligase-7 (CRL-7) [141]. Insulin receptors can be negatively controlled by phosphatase and tensin homolog (PTEN) and tyrosine-protein phosphatase nonreceptor type 1 (PTPN-1) [142, 143].

IRS-1 and IRS-2 tyrosine phosphorylation are responsible for the insulin signaling cascade, but the serine and threonine residues are phosphorylated before, during, and after insulin activation [130, 144]. IRS-1 has about 200 serine/threonine residues, of which around 30 are known to be phosphorylated [145, 146]. Of all the serine and threonine residues, S^307^ is the most investigated because it is implicated in physiological and pathological conditions, including insulin resistance, hyperinsulinemia, obesity, and ER stress [130, 147–149]. It is essential to note that s^307^ contributes to insulin resistance, but its phosphorylation is also critical in the physiological insulin cascade [150]. In the study by Copps et al. [150], this remained the case when s^307^ was replaced with Ala-^307^; it equally did not show hypersensitivity, but it did promote significant insulin resistance in the mice used [150]. Insulin resistance may worsen due to hyperinsulinemia, which then compounds the problem of serine and threonine phosphorylation on IRS-1[151]. To date, there is no holistic understanding of insulin signaling cascade and regulatory mechanisms, the challenge in particular being the high serine and threonine residues on IRS-1.

In pathological conditions such as diabesity, multiple sites of IRS-1 containing serine and threonine may be phosphorylated by many kinases, for example, pelle-like kinase/interleukin-1 receptor-associated kinase (mPLK), lipid/inflammatory stimulated JNK, an inhibitor of nuclear factor kappaB kinase beta (IKKB), PKC, and sympathetic-activated protein-coupled receptor kinase 2 [130] and ER stress [152]. ER stress is part of the physiopathological processes that feature in beta cell failure, leading to the disease progression.

ER stress is believed to cause insulin resistance via the activation of JNK, which leads to the inhibition of IRS-1 by phosphorylating the serine residue [153]. The activated JNK causes insulin resistance in obesity and possibly in diabesity in four ways: inhibition of IRS-1 phosphorylation directly, the inhibition of PPAR*α*-FGF21 axis hormones, induction of cytokines and inflammation, and enhanced metabolic and adipogenesis efficiency [153, 154]. Also, Liang et al. [152] have shown that ER stress leads to phosphorylation of IRS-1 at S^307^ and reduced Akt phosphorylation via JNK. In contrast, a study by Brown et al. [155] has shown that ER stress does not affect Akt phosphorylation at S^473^ and tyrosine IRS-1 phosphorylation. During ER stress, there is a reduction in the availability of matured insulin receptors and, at the same time, inhibition of phosphorylated Akt. It is important to note that the half-life of insulin receptors increases during ER stress [155].

ER stress and IRS exhibit a complex association, so it is necessary to determine if IRS affects ER stress in a feedback mechanism. The importance of IRS-1 or IRS-2, particularly under ER stress conditions, has not been thoroughly investigated in diabesity. Moreover, it remains unclear whether there is a phenotypic difference between IRS-1 and IRS-2 in diabesity during ER stress.

## 9. ER Stress and Nicotinamide Adenine Dinucleotide Phosphate (NAD(P)H) Oxidase

**Figure 3:**
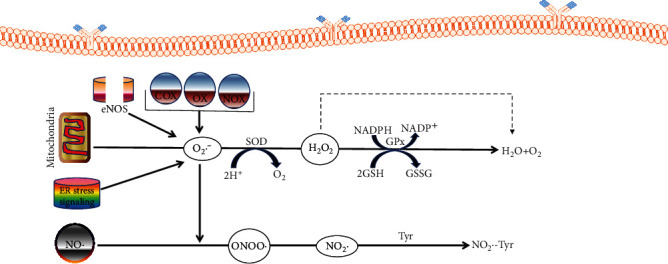
The sources of superoxide anion. This figure shows how superoxide anion is obtained from various sources that generate hydrogen peroxide (H_2_O_2_) by superoxide dismutase (SOD). H_2_O_2_ can be catalyzed to H_2_O. NO can react with superoxide anion to produce reactive nitrogen species (ONOO) that could be changed into NO_2_, reacting with tyrosine residues to produce nitrotyrosine. These factors result in the reduced availability of NO and also lead to endothelial impediments.

A transmembrane enzyme known as NAD(P)H oxidase (NOX) generates reactive oxygen species (ROS) as its major function. It consists of many subunits, among which are 2-activator subunits (NOXA1 and p67^phox^), 7-NOX, 2-DUOX-specific maturation factors, and 2-organizers (p47^phox^ and NOXO1) [156–158]. Specifically, NADPH oxidase families such as NOX-1, NOX-2, NOX-3, NOX-4, and NOX-5 generate superoxide anion, as well as hydrogen peroxide as a downstream metabolite [156]. NOX oxidase has an electron transfer system consisting of the C terminal cytoplasmic region and the N terminal. NOX facilitates the movement of electrons from cytosolic NADPH via flavin adenine dinucleotide (FAD), which leads to superoxide anion (O_2_^−^) production in the cytosol [34]. NOX, mitochondria, xanthine oxidase (OX), uncoupled eNOS, cytochrome P-450 oxygenase, and cyclooxygenase (COX) are some sources of ROS, as depicted in Figure 3.

NOX-2 is often colocalized with p22^phox^ in the membrane of intracellular vesicles, but the phosphorylation of p47^phox^ results in the translocation of p47^phox^-p67^phox^ from the cytoplasm to the plasma membrane. The p47^phox^/p67^phox^ interacts with p22^phox^, followed by translocation of p40^phox^; this sequence of events activates the NOX-2 [159, 160]. The activated NOX-2 results in the fusion of the vesicle to the plasma membrane releasing superoxide anion [156]. Some studies reported that NOX-2 and NOX-4 produce hydrogen peroxide (H_2_O_2_) only; however, other research has shown the production of superoxides (O^2-^) as well [161]; the different results perhaps being attributable to the methods used or experimental conditions. NOX-2 has phagocytic and electron activities associated with phagosomes and proteases [162–164]. The expression and stimulation of NOX families (especially NOX-2) are associated with amplified Ca^2+^ release [156] and UPR stimulation [165]. The two lipids (cholesterol and 7-ketocholesterol) induce Ca^2+^ release and CHOP via ER-receptors called the inositol-1,4,5-triphosphate receptor type-1 (IP3R-1) and Ca^2+^/calmodulin-dependent kinases-II (CaMK-II), respectively. The activation of these ER-receptors leads to ROS production, especially via the activation of NOX-2. The ROS produced by NOX-2 is thought to be sustained by CHOP and eIF2*α* activation, serving as a feedback mechanism during ER stress [165]. The activation of ER stress through CHOP leads to more significant intracellular Ca^2+^ release and eventually causing more NOX-2 stimulation [165]. The ROS generated by NOX-2 is implicated in physiological processes such as cellular differentiation, proliferation, cytoskeletal organization, and migration [166].

The ER-localized NOX-4 has been found to lead to eNOS uncoupling and increased ROS [98]. Under prolonged ER stress, the JNK and ASK-1 signaling cascade is activated via IRE-1, leading to ER dysregulation and apoptosis [167]. The lack of NOX-4 hindered the transcription of many UPR markers, such as CHOP and Bax, associated with cell death initiation due to the activation and phosphorylation of JNK and ASK1, a signaling cascade of IRE1 activation [168]. The disulfide isomerase (PDI) enzyme on the ER is associated with NOX-4 and leads to reduced phosphorylation of Akt [169], which affects the survival of the cells. The physical association between p22^phox^ oxidase and PDI has been demonstrated in macrophages and neutrophils [170, 171]. The PDI-NOX-induced ROS downstream function remains unclear but may be linked to phagocytosis mediated by the ER [164]. ER-localized NOX-4 mediates oxidation; thus, inactivation of protein tyrosine phosphatase (PTB1B) during UPR, PTP1B may trigger IRE1-dependent signaling, an essential target for NOX-4 [172].

The generation of ROS is likely to happen during ER stress reaction and the orchestrated three-arm sensors. Also, an increase in ROS generation from the stimulated NOX may lead to diabesity. NOX-1, NOX-2, and NOX-4 oxidases are associated with ER stress redox signaling and metabolism for proapoptotic or prosurvival consequences. NOX-mediated ROS (hydrogen peroxide and superoxide) generation may stimulate other enzymatic systems to generate ROS [173, 174]. For instance, NOX generates ROS, which leads to oxidation of BH_4_, a crucial factor for NO production in eNOS [175–178]. NOX has recently emerged as a critical oxidase mechanism that underlies oxidative stress in diabetic complications [179, 180] and diabesity [35]. Animal models of type 1 and type 2 diabetes have also registered NOX activation [181]. Prolonged activation of NOX has been associated with endothelial dysfunction in diabetes and diabesity. Notably, a few of the critical implications of NOX induction are stimulating several other oxidase mechanisms called NOX-dependent ROS production to maintain oxidative stress; these include ER stress, eNOS uncoupling, mitochondria, OX, cytochrome P-450 oxygenase, and COX. The functions of NOX families in association with ER stress are not fully elucidated.

ROS production is increased through the NOX family enzymes, primarily through the NOX-1, NOX-2, and NOX-4 isoforms under ER stress [182, 183]. NOX-1 [182], NOX-2 [165], and NOX-4 are the three NOX isoforms hitherto confirmed to have been involved in ER stress [95, 165, 168, 184]. Camargo et al. [182] demonstrated that NOX-1 and NOX-4 are responsible for the irreversible oxidation and phosphorylation of PERK and IRE-1, respectively. NOX involves a different process in ER stress modulation, involving NOX-1-regulated PERK and NOX-4-regulated IRE-1. The plasma membrane is localized with NOX-1 and NOX-2, while the ER is localized with NOX-4, leading to increased ROS production in a cell.

During ER stress, hyperoxidation of the ER environment occurs, which initiates a UPR signaling cascade, resulting in the transfer of electrons from NADPH. These have a redox effect on ER and promote enhanced consumption of glutathione and thioredoxin reductase. Zeeshan et al. [185] identified that numerous external agents might stimulate intracellular ROS production, which decreases the antioxidant defense mechanism that can lead to an increase in oxidative stress and ER stress. Therefore, to achieve the therapeutic potential of targeting NOX isoforms and downstream oxidase systems to prevent and treat diabesity, innovative, reliable, and specific NOX isoform inhibitors are required. Also, the design of new approaches targeting other sources of ROS is crucial in diabesity.

## 10. Caveolin 1 (Cav-1) and eNOS

Caveolae are cell surface plasma membrane invaginations of 50 nm to 100 nm in diameter. They play a critical role in signal transduction, transcytosis, mechanosensation, mechanoprotection, maintenance of plasma membrane integrity, endocytosis, and lipid homeostasis [186]. They were once called simple membrane structures and are now regarded as more complex systems. The caveolae's primary membrane proteins contain caveolins that invaginate the cell membrane. The caveolins are divided into three types, i.e., caveolin-1, caveolin-2, and caveolin-3 (Cav-1/2/3) [187]. The protein Cav-1 is oligomerized in the ER, after which it translocates to the Golgi body, where it may interact with cholesterol molecules before final release to the cellular membrane [188]. In most cell types, Cav-1 is expressed and crucial for the biosynthesis of caveolae. Cav-1 is usually expressed in endothelial cells, adipocytes, fibroblasts, and pneumocytes, along with Cav-2, which indicate they both play an essential function in the caveolar [189]. A total absence of caveolae results in loss of Cav-1 [190]. Other proteins have also been identified that play a crucial function in developing caveolae, for example, Cavin 1 to 4, pacsin2, and Eps 15 homology domain-containing protein 2 (EHD2) [191, 192].

**Figure 4:**
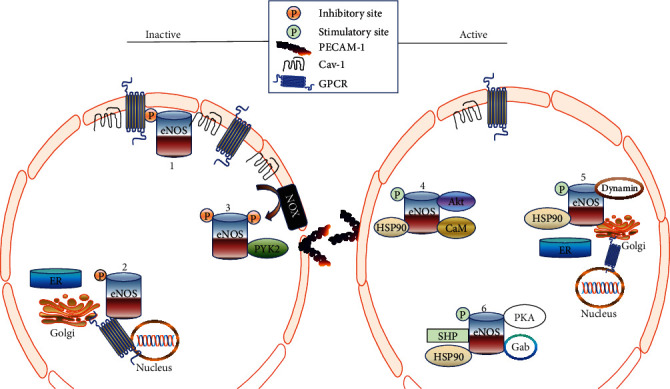
eNOS/Cav-1 interaction; it may be seen that eNOS is a homodimer limited to the Golgi apparatus and plasma membrane caveolae. In an inactive state, the caveolae protein is coupled to Cav-1, which reduces its action. Furthermore, when eNOS is phosphorylated by protein kinase C on Thr^495^, it inhibits contact with CaM. Inhibition of the enzyme can be achieved in a situation of oxidative stress caused in the aftermath of proline-rich tyrosine kinase 2- (PYK-2-) induced tyrosine phosphorylation of eNOS. Owing to cellular activation of eNOS, which is split from Cav-1, CaM can bind to the eNOS at Ser^1177^, thanks to Thr^495^ dephosphorylation, and produce EDRF.

eNOS [193], G-proteins [194], protein kinase A (PKA) [195], protein kinase C (PKC) [196], and many other receptors are identified in associated caveolae-enriched signaling molecules. Cav-1 has been proposed to bind and inhibit them via its caveolin scaffolding domain (CSD), a preserved amphipathic area for the development of caveolae and signaling cascade [194, 197, 198]. eNOS has received significant interest among these caveolae-located signaling molecules, thanks to its crucial vascular homeostasis effects [86, 199]. An investigation has shown that most eNOS dwell in endothelial cell caveolae [200, 201]. The findings underline the essential role of endothelial caveolae in managing eNOS activation, as depicted in Figure 4. eNOS has been linked with Cav-1 in nonactive endothelial cells, which hinders the calcium calmodulin complex (CaM) from binding to eNOS [202]. The interaction between eNOS and Cav-1 prevents the electron transfer from NADPH to eNOS.

The M3-muscarinic acetylcholine receptor (M3) is a G protein-coupled receptor (GPCR) coupled to the Gq heterotrimeric protein through phospholipase C signaling cascades to produce cytosol calcium. For instance, ATP, bradykinin, endothelin-1, histamine, thrombin, ang II, and acetylcholine bind to M3 receptors to activate phospholipase C enzymes. The activation of these enzymes leads to the cleavage of phospholipid (phosphatidylinositol-4,5-bisphosphate) to produce inositol-1,4,5-trisphosphate (IP3) and diacylglyceride. The IP3 produced leads to the activation of inositol-1,4,5-trisphosphate receptors (IP3Rs) localized on the ER. The increase in IP3 produces a calcium signal within the endothelial cells, which takes a calcium wave, resulting in the release of calcium. The calcium-binding site, also known as the EF-hand domain, contains the N lobe and the C lobe calmodulin. The EF-hand domain is a polypeptide structure capable of detecting calcium. Calcium binds to the calmodulin to form a complex compound called the calcium-calmodulin complex (Ca^2+^-CaM). eNOS dissociates itself from Cav-1 and then merges with Ca^2+^-CaM. This allows the flow of electrons from NADPH and subsequently releases NO from eNOS [203, 204]. eNOS moves from the cell membrane to the Golgi complex, thanks to a higher cytosolic Ca^2+^ concentration, and is entirely activated. The production of NO leads to a reunion between eNOS and Cav-1, thereby halting the signaling cascade [205]. In vivo and in vitro studies have shown that eNOS can bind to Cav-1 and hinder NO synthesis [206, 207]. It is understood that overexpression of Cav-1 is linked to reduced acetylcholine-induced NO generation and vasodilation in patients with insulin resistance and type 2 diabetes [208]. Type 2 diabetic patients have shown downregulation of eNOS and Cav-1 expression in vascular endothelial cells [209]. However, it is unknown if there is either an increase or decrease in Cav-1 expression in diabesity, as seen in type II diabetes. A study by Rodrigues et al. [210] has shown that caveolae dismantled with methyl-*β*-cyclodextrin (m*β*cd) treatment cause diminished relaxation by acetylcholine isolated aorta of the rat. Also, Al-brakati et al. [211] have demonstrated that caveolar disturbance results in decreased NO in femoral arteries. A study by Shamsaldeen et al. [212] has shown a decreased expression of eNOS and Cav-1 in the streptozotocin type I diabetic rat model. These disturbances in caveolae can decrease the levels of Cav-1 associated with eNOS, thereby undermining vascular function. The decrease in eNOS and Cav-1 expression in aortic endothelial cells from STZ-diabetic rats can inhibit PI3K/Akt cascades since wortmannin prevents eNOS and Cav-1 from moving to the cell membrane [213]. Insulin is known to stimulate a PI3K/Akt cascade, such that it allows eNOS and Cav-1 to move toward the cell membrane. Also, insulin triggers eNOS palmitoylation via Golgi palmitoyltransferase, enabling the acetylation of eNOS and Cav-1, which both of them translocate to the cell membrane [214]. The palmitoylation of eNOS and Cav-1 has been found to enhance the binding to the cell membrane nearly 10-fold, a process needed to maximize eNOS activity [215].

It is interesting to note that any metabolic disorders such as obesity, insulin resistance, diabetes, and diabesity may cause a change in the structural integrity of caveolae or Cav-1 concentration, leading to vascular dysfunction. Therefore, there is a need to determine the structural and molecular elements of how eNOS and Cav-1 influence each other's role in endothelial cells and how endothelial dysfunction leads to diabesity because of their deficiency. Ultimately, further examination and clarification are likely to yield more insights into ways of reversing the process of restoring vascular homeostasis due to diabesity.

## 11. The Prospect of ER Stress and Associated Cellular Events

ER stress may lead to cellular death; it has been associated with the instigation and advancement of many diseases. The mitigation of ER stress and its residents has opened up new therapeutic frontiers for interventions in associated diseases [216]. Toth et al. [217] reported that ER stress and other cellular components play a vital role in survival and apoptosis, making them the best target for therapeutic intervention. The circumstances involving proapoptotic events in ER stress are associated with many pathways, including TRAF-2, ASK-1, CHOP, and JNK, thus developing a therapeutic approach that aims for a particular apoptotic path may not be sufficient to preserve cells [28]. Therefore, drug innovations require a holistic approach to comprehend the interrelationship between pathways involved in apoptosis due to ER stress.

The proapoptotic pathways and the arms of the UPR signaling pathways are extensively expressed during ER stress; therefore, averting their action could cause unprecedented adverse effects on the subject in question. Much research is desirable to elucidate the character of ER stress completely and the best therapeutic approaches to use within the connecting system. ER stress-associated molecular mechanisms and gene regulation that induce and advance cellular apoptosis are crucial for discovering novel molecular markers for future drug innovations in diabesity. Similarly, it is imperative to look at the contributions of noncanonical pathways in relation to diabesity during ER stress.

## 12. Conclusion

Endoplasmic reticulum homeostasis is critical in maintaining cellular functions. Sustained ER stress is associated with several metabolic disorders, including diabesity, mainly owing to the interplay of chronic hyperglycemia and hyperlipidemia. The ER stress response is now recognized as a converging molecular mechanism connecting insulin resistance, lipid metabolism distress, and oxidative stress to endothelial dysfunction, and cell death. ER stress also results in the accumulation of misfolded proteins countered by UPR associated with the activation of proinflammatory and proapoptotic pathways. Thus, understanding ER stress and its mechanisms, including homeostatic regulators such as the UPR, could help identify molecular targets for diabesity treatment.

## Data Availability

This being a review article, no data was generated during the preparation of this manuscript.
