# Correction to “Potential Roles of Endoplasmic Reticulum Stress and Cellular Proteins Implicated in Diabesity”

**DOI:** 10.1155/omcl/9817849

**Published:** 2026-01-29

S. Mustapha, M. Mohammed, A. K. Azemi, et al., “Potential Roles of Endoplasmic Reticulum Stress and Cellular Proteins Implicated in Diabesity,” *Oxidative Medicine and Cellular Longevity* 2021 (2021): 8830880, https://doi.org/10.1155/2021/8830880.

In the article titled, “Potential Roles of Endoplasmic Reticulum Stress and Cellular Proteins Implicated in Diabesity,” there was an error in the Acknowledgments section where the statement “Funding for this work was provided by the Ministry of High Education, Malaysia, via the Fundamental Research Grant Scheme (203/PPSP/6171215)” is incorrect. The corrected section appears below:

Acknowledgments

This research was funded by the Fundamental Research Grant Scheme [FRGS/1/2018/SKK08/USM/02/11], Ministry of Higher Education, Malaysia.

We apologize for this error.

